# Accelerating cardiac diffusion tensor imaging with deep learning-based tensor de-noising and breath hold reduction. A step towards improved efficiency and clinical feasibility

**DOI:** 10.1016/j.jocmr.2025.101971

**Published:** 2025-11-08

**Authors:** Michael Tänzer, Andrew D. Scott, Zohya Khalique, Maria Molto, Ramyah Rajakulasingam, Ranil De Silva, Dudley J. Pennell, Pedro F. Ferreira, Guang Yang, Daniel Rueckert, Sonia Nielles-Vallespin

**Affiliations:** a Imperial College London, London, United Kingdom; b Royal Brompton and Harefield Hospital, London, United Kingdom; c Technische Universität München, München, Germany

**Keywords:** Cardiac MRI, Deep learning, Diffusion tensor imaging, MRI

## Abstract

**Background:**

Cardiac diffusion tensor imaging (cDTI) non-invasively provides unique insights into cardiac microstructure. Current protocols require multiple breath-hold repetitions to achieve an adequate signal-to-noise ratio, resulting in lengthy scan times. The aim of this study was to develop a cDTI de-noising method that would enable the reduction of repetitions while preserving image quality.

**Methods:**

We present a novel de-noising framework for cDTI acceleration centered on three fundamental advances as follows: (1) a paradigm shift from image-based to tensor-space de-noising that better preserves structural information, (2) an ensemble of Vision Transformer-based models specifically optimized for tensor processing through adversarial training, and (3) a sophisticated data augmentation strategy that maximizes training data utilization through dynamic repetition selection.

**Results:**

Our approach reduces scan times by a factor of up to 4 while achieving a 20% reduction in cDTI maps errors over existing de-noising methods (fractional anisotropy errors 0.09 vs 0.07) and preserving anatomical features such as infarct characterization and transmural cardiomyocyte orientation patterns. Crucially, our proposed method succeeds in clinical cases where other algorithms previously failed.

**Conclusion:**

This demonstrates substantial improvements in cDTI acquisition efficiency, achieving up to four-fold scan time reduction (3–5 breath-holds) while maintaining diagnostic accuracy across diverse cardiac pathologies.

## Graphical abstract

**Figure:**
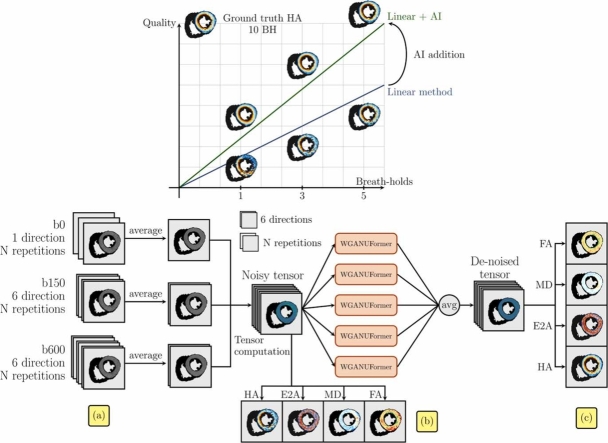

## 1 Introduction

Cardiac diffusion tensor imaging (cDTI) stands out as a unique imaging technique capable of non-invasively probing the cardiac microstructure in living subjects [1]. By depicting the pattern of diffusion of water molecules within the tissue, cDTI provides information about the structure in which these water molecules are embedded [2], [3], [4], [5]. The technique acquires multiple diffusion-weighted images (DWI) with varying sensitivities (b-values) and directions to capture the anisotropic nature of myocardial water diffusion. From these measurements, diffusion tensors—3 × 3 symmetric matrices modeling water diffusion as an ellipsoid—are computed for each voxel. The tensor’s eigenvalues and eigenvectors yield quantitative maps that characterize different aspects of cardiac microstructure: mean diffusivity (MD) represents the average magnitude of water diffusion, fractional anisotropy (FA) reflects tissue organization, while helix angle (HA) and second eigenvector angle (E2A) describe cardiomyocyte and sheetlet orientations, respectively [2], [3], [6]. This microstructural characterization has been proven to be a useful tool for clinical research, phenotyping numerous cardiomyopathy, myocardial infarction, and congenital heart disease patient populations [7], [1], [8], [9], [10], [11], [12], [13], [14].

**Fig. 1:**
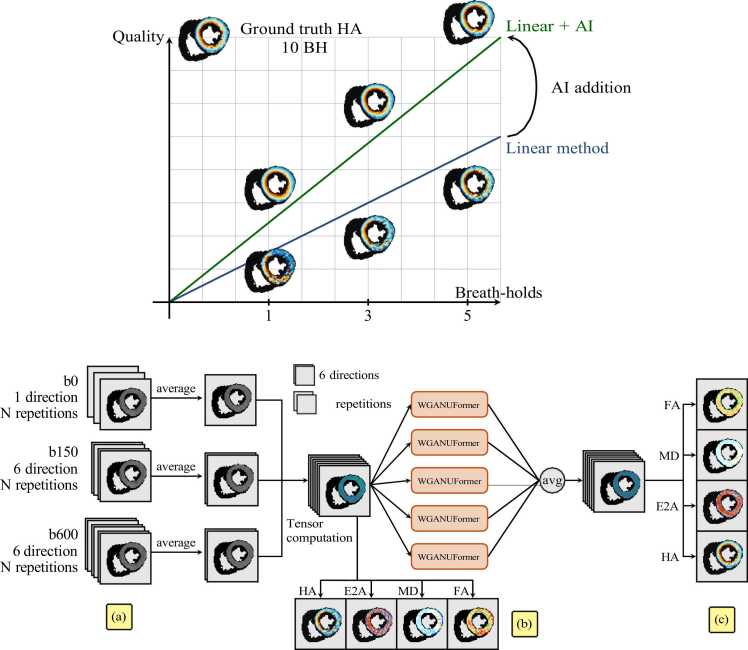
Proposed deep learning framework with an example of how our method enhances image quality for reduced acquisition durations (top). To improve the signal-to-noise ratio, the original input data (a) undergoes averaging across multiple repetitions. Employing a least squares tensor fitting approach, the averaged data is processed to generate initial diffusion tensor estimates, which may contain noise artefacts. These preliminary tensors can be used to compute noisy cDTI-derived maps (b). Subsequently, an ensemble of machine learning algorithms is applied to refine and de-noise the diffusion tensors. The resulting tensors allow for the computation of higher-quality cDTI maps (c). *cDTI* cardiac diffusion tensor imaging

The long acquisition time is currently the main factor limiting the clinical translation of cDTI. cDTI requires the acquisition of DWIs in at least six directions, plus images with no or minimal diffusion weighting called *b*_0_. One cDTI STEAM repetition containing one *b*_0_ image plus 6 DWIs, including preparation pulses, requires 18 heartbeats. At least eight repetitions need to be acquired to achieve sufficient SNR, which results in approximately 8 min per slice at one cardiac phase [9], [8], [7]. When assessing cardiac microstructure dynamics, data is acquired at least at two different time points (systole and diastole), which leads to 16 min per slice. For whole-heart coverage (∼ 12 slices), that would result in 192 min, hindering the protocol’s clinical applicability. Lowering the number of cDTI repetitions improves efficiency but inherently degrades SNR and image quality (Fig 1, top).

Noise reduction techniques aim to extract the underlying signal from data contaminated by unwanted perturbations. While some models are developed for specific noise distributions [15], others employ distribution-agnostic approaches, potentially offering greater versatility in addressing real-world signal interference [16]. The field of deep learning has significantly advanced de-noising methods, with applications spanning various domains, including photography [17], magnetic resonance imaging (MRI) [18], [19], computed tomography (CT) [20], audio processing [17], and point cloud data [21]. In the realm of image de-noising, notable approaches include Batson et al.’s [16] self-supervised U-Net model for blind de-noising, and Park et al.’s [22] generative adversarial network (GAN)-based method for unpaired CT data. Recent advancements have seen the application of Vision Transformers to image restoration tasks [23], [24], [25]. The UFormer [26] architecture has gathered attention as it inherits key advantages of Transformer models (e.g., adaptable filters) while restricting the self-attention process to a limited patch. This architecture is particularly beneficial for our application, as image corruptions in cDTI predominantly manifest as localized patterns that can arise from both random noise and systematic artefacts, where UFormer excels with its window-based self-attention mechanism. The LeWin Transformer blocks efficiently capture these local noise patterns while the hierarchical structure still maintains awareness of the global anatomical context, offering an optimal balance between noise removal capability and computational efficiency.

While deep learning approaches have demonstrated potential for restoring signal quality from reduced-repetition acquisitions, current solutions fall short of clinical requirements. In cDTI, Phipps et al. [27] used a residual-learning approach to de-noise the diffusion-weighted images prior to the tensor calculation to reduce the number of acquisitions required to produce cDTI maps. In previous work, [28] showed how a U-Net-based model can be used successfully to predict de-noised tensors directly from noisy images. However, this approach can produce cDTI-derived maps that deviate from full-repetition references in ways that compromise diagnostic accuracy, particularly when imaging unusual cardiac anatomies. This deviation stems from two key issues: first, the natural scarcity of pathological cases in the training data biases these models toward producing “normal-looking” outputs, effectively smoothing out real anatomical abnormalities. Second, the strong spatial smoothing characteristics inherent to U-Net architectures, while effective for noise reduction, can blur or eliminate subtle pathological features that are critical for diagnosis.

### Contribution

We present three fundamental advances to overcome the challenges we face when reducing the number of breath-hold repetitions in cDTI as follows:

1. A paradigm shift from existing image-based models to diffusion tensor-space de-noising that better preserves structural information.
2. An ensemble of Vision Transformer-based models [29] specifically optimized for tensor processing.
3. A sophisticated data augmentation strategy that leverages dynamic repetition choice to better utilize the available training data.

Fig. 1 depicts a schematic representation of our contribution. As demonstrated in the Results section, these advances enable our model to not only produce superior diffusion tensor maps from reduced-repetition data but crucially maintain robustness in pathological cases where state-of-the-art methods fail.

## 2 Methods

### 2.1 Data acquisition

The research protocol for this study received approval from the National Research Ethics Service. All participants provided written informed consent prior to their involvement. Data acquisition was performed using two MRI systems: a Siemens Skyra 3T scanner and, in more recent cases, a Siemens Vida 3T scanner (both from Siemens Healthineers, Erlangen, Germany). The imaging sequence employed was a diffusion-weighted stimulated echo acquisition mode (STEAM) single-shot echo planar imaging (EPI) protocol. This sequence featured a reduced phase field-of-view and fat suppression, with the following parameters: TR = 2 cardiac cycles, TE = 23 ms, parallel imaging acceleration factor (sensitivity encoding [SENSE] or generalized autocalibrating partially parallel acquisition [GRAPPA]) R = 2, and an echo train duration of 13 ms. Images were acquired at a spatial resolution of 2.8 × 2.8 × 8.0 mm^3^. The mean number of repetitions for each acquisition varied across different b-values: 12 ± 2.0 for b_0_ images, 10 ± 2.2 for b = 600 s/mm^2^ images, and 2 ± 0.6 for b = 150 s/mm^2^. Data were acquired in either the diastolic pause (49%, n = 368) or end-systole (51%, n = 376).

The study encompassed a total of 744 cDTI datasets, comprising a diverse cohort of 197 healthy volunteers (26%) and 547 patients (74%). The patient group included individuals with various cardiac conditions. Detailed demographic information can be found in Table 1. Overall, this study uses the same data as Ferreira et al.’s [28] work.

**Table 1:** Volunteers distribution across different cardiac conditions^a^

Condition	Total Patients	Percentage (%)
AMYLOID	31	4.2
DCM	45	6.0
Fabry’s Disease	11	1.5
HCM G + P-	48	6.5
HCM	66	8.9
hDCM	4	0.5
Healthy	197	26.5
Acute MI	246	33.1
Marfan’s Syndrome	7	0.9
rDCM	89	12.0
Total	744	100.0

*DCM* dilated cardiomyopathy, *HCM* hypertrophic cardiomyopathy, *MI* myocardial infarction

a Data are number of patients for different conditions (absolute values) and their relative percentages (%).

### 2.2 Data preparation

Prior to tensor calculation, all diffusion images underwent visual inspection. Images exhibiting significant motion artefacts were excluded from further analysis. The retained images were then manually thresholded to remove background noise and co-registered using an advanced multi-resolution rigid sub-pixel translation algorithm that resamples using a cubic B-spline [30]. Subsequently, the left ventricular (LV) myocardium was manually delineated, excluding the papillary muscles. Finally, the diffusion-weighted images were averaged, and the tensors were computed.

Datasets containing all repetitions were utilized to generate reference tensor data for each subject using a Linear Least Squares (LLS) fitting approach [31], as this was the method originally employed to calculate cDTI maps from the data at the time of acquisition. An in-house-developed Python-based tool was employed for computing the diffusion tensors, with its accuracy validated against established post-processing software [3]. These tensors served as the foundation for generating cDTI maps, which were used as the reference standard for all comparative analyses in this study.

To investigate the feasibility of reducing acquisition time, we examined the impact of decreasing the number of repetitions on cDTI map quality. Three new datasets were created with decreasing numbers of repetitions:

• 5BH: Four repetitions each of b_0_ and b_600_, with one b150 repetition, requiring 5 breath-holds (BH).
• 3BH: Two repetitions each of b_0_ and b_600_, with one b150 repetition, requiring 3 breath-holds.
• 1BH: Single repetition of b_0_ and b_600_ only, requiring 1 breath-hold.

For training purposes, we incorporated alternative repetition sampling strategies to more efficiently use all available data, effectively expanding the training dataset. Instead of using only the first *N* repetitions for each acquisition, we created multiple training samples from each scan by selecting three distinct subsets: the first *N* repetitions, the central *N* repetitions, and the final *N* repetition, effectively tripled our training data. This sequential sampling strategy was chosen because it better reflects realistic clinical acquisition scenarios where repetitions are acquired consecutively in time. The first, middle, and last repetition subsets capture temporal variations in patient physiology, breathing patterns, and potential motion artifacts that occur during sequential breath-hold acquisitions, making the trained model more robust to the systematic variations encountered in prospective clinical use. For testing, we maintained the use of only the first *N* repetitions to closely approximate a prospective acquisition scenario. Additional standard data augmentation techniques, including random rotation and content-aware cropping, were applied to further enrich the training dataset for deep learning model development.

### 2.3 Tensor de-noising with deep learning

We propose WGANUformer (WGUF), a novel deep-learning approach that leverages cutting-edge architectures for tensor refinement. Our method combines a UFormer-based [26] generator with adversarial learning and sophisticated data augmentation strategies to enhance the quality of cDTI tensors derived from reduced-repetition acquisitions. The approach introduces the following two key innovations: (1) a tensor-specific adaptation of the UFormer architecture that handles the unique characteristics of cardiac diffusion imaging and (2) an efficient data augmentation strategy that maximizes the utility of limited cardiac imaging datasets. The model is trained using a PatchGAN [32] discriminator and Wasserstein loss function to ensure stable convergence and high-quality outputs.

#### 2.3.1 Data augmentation strategy

To address the challenges of limited medical imaging data, we developed a targeted augmentation approach. The core of this strategy involves dynamic repetition sampling, where we generate three distinct versions of each acquisition by sampling the initial, central, and final M repetitions. This effectively triples the available training data while capturing realistic variations in acquisition timing. Additionally, we apply standard spatial augmentations, including random rotation and content-aware cropping, ensuring the model learns invariant features. This combined approach proves particularly valuable for tensor refinement, as it introduces realistic acquisition variations while preserving the underlying cardiac structure and maintaining realistic noise patterns.

#### 2.3.2 Model architecture

The WGANUformer architecture builds upon the UFormer framework [26], which represents state-of-the-art performance in image enhancement tasks. Building upon this foundation, we developed an approach that extends the UFormer framework by integrating it into an adversarial learning paradigm. Our proposed WGANUformer (WGUF) combines a UFormer-based generator with a PatchGAN [32] discriminator. The training process implements a Wasserstein loss function with gradient penalty, following the principles outlined by Arjovsky et al. [33], as this approach has demonstrated superior stability and convergence properties compared to traditional GAN formulations. The resulting model comprises approximately 40 million parameters.

To further enhance performance and robustness, we employ an ensemble strategy, averaging the predictions of five independently initialized and trained instances of the WGUF model. This bagging ensemble formulation helps reduce module variability due to aleatoric uncertainty. The resulting architecture maintains the computational efficiency of the UFormer model while adding the perceptual quality benefits of adversarial training.

To systematically evaluate our approach, we first established a baseline using the model from Ferreira et al. [28]. Their model employs a U-Net [34] convolutional neural network with a symmetric encoder-decoder structure comprising six levels in each branch. We re-trained this model on our dataset while maintaining the original architecture, training procedure, hyperparameters, and pre-processing steps. This baseline implementation contains 31 million trainable parameters.

To understand the contribution of each component of our approach, we systematically developed several variants by progressively introducing modifications to the baseline model. Throughout these experiments, the main architecture and training hyperparameters remained constant to ensure fair comparison. The following variants were developed to address specific challenges in cDTI processing, particularly the multi-channel nature of tensor data and limited dataset size:

1. Baseline U-Net convolutional network for image-to-tensor computation.
2. U-Net network with channel normalization (UN+CN): the target tensors underwent standardization using channel-specific statistics. This normalization procedure helps the model by equalizing the scale across channels, which originally exhibited significant range disparities.
3. U-Net network adapted for tensor refinement (UN+T2T): the input modality was shifted from images to tensors, re-framing the learning objective as a tensor refinement task. This modification allowed for the incorporation of residual learning, promoting faster convergence and improved overall performance. Both input and target tensors were scaled by a predetermined factor.
4. UN+CN adapted for tensor refinement (UN+CN+T2T): similar to UN+T2T, but employing z-score standardization for both input and target tensors.
5. UN+CN+T2T with augmented datasets (UN+CN+augT2T): the training process incorporated diverse sampling strategies (initial M repetitions, central M repetitions, final M repetitions). This approach maximized the utility of the limited dataset, effectively tripling the available training samples, albeit with some redundancy.

#### 2.3.3 Input and output

All models in our study, including both the proposed WGANUformer and the baseline variants, generate diffusion tensors represented at each pixel by six unique elements (entries in the 3 × 3 symmetric diffusion matrix). Positive semi-definiteness of the matrix is not enforced. To optimize processing, the spatial dimensions of both input and output data were cropped and padded to 128 × 128 pixels, yielding a six-channel 128 × 128 output image.

Inputs varied between diffusion-weighted images with either 7 (1 b_0_ + 6 b_600_) or 13 (1 b_0_ + 6 b_600_ + 6 b_150_) channels, and diffusion tensors represented as six-channel images. Diffusion-weighted images underwent global normalization to the range [0, 1], with non-cardiac regions zeroed out. For tensor inputs, two normalization strategies were explored: scaling by a factor of $\frac{1}{500}\frac{mm^{2}}{s}$ (empirically determined to constrain most values within [ − 1, 1]) and applying channel-specific z-score normalization based on training set statistics. Prior to generating cDTI maps, we reversed the normalization process.

The dataset was partitioned into training, validation, and test subsets following an 80:10:10 ratio using patient-level splitting to prevent data leakage between sets. The partitioning was performed using a fixed random seed to ensure reproducibility, with patients randomly assigned to each subset while maintaining the specified proportions. To ensure experimental consistency, the same partitioning was maintained across all experiments, with validation set performance used for model selection and hyperparameter tuning.

#### 2.3.4 Training procedure

The neural networks were trained using mean absolute error as the optimization criterion, with all models initialized randomly and trained from scratch without transfer learning over 500 epochs with a batch size of 8 images. The U-Net-based architectures were optimized using the Adam algorithm with the following hyperparameters: learning rate of 10^−4^, beta1 = 0.9, beta2 = 0.999. The WGANUformer employed the AdamW optimizer as described by Loshchilov et al. [35], with learning rate = 10^−4^, beta1 = 0.9, beta2 = 0.999, and weight decay alpha = 0. These configuration choices were determined through empirical evaluation in preliminary studies. The computational experiments were conducted on a high-performance Linux workstation running Ubuntu 18.04, equipped with an Intel i7–10700k central processing unit, 64 GB of random access memory, and an NVIDIA RTX 3080 GPU (NVIDIA, Santa Clara, California). The software environment consisted of Python 3.8 and PyTorch 1.9. The total computational time amounted to 215 GPU hours, with an estimated carbon footprint of 30 kg CO_2_eq. For performance evaluation, all reported metrics were calculated using an independent test set that was not exposed to the models during the training or validation phases. The specific model checkpoint used for evaluation was selected based on the lowest loss achieved on the validation set.

### 2.4 cDTI maps comparison

HA maps represent angular information within the range of −90^∘^ to 90^∘^. As defined in [3], angles differing by 0^∘^ or 180^∘^ indicate vectors with identical orientation and should be treated as equivalent. Conversely, angles separated by 90^∘^ represent the maximum possible directional difference. Consequently, the Mean Absolute Angle Error (MAAE) for HA is calculated as

(1) $$\mathrm{MAAE}\left(x,y\right)=\left\{\begin{array}{c} |x-y|,\;\mathrm{if}\;|x-y|<90^{{}^{\circ}}\;\\ 180^{{}^{\circ}}-|x-y|,\;\mathrm{otherwise}\; \end{array}\right)$$

For the scalar quantities MD and FA, the Mean Absolute Error (MAE) between the de-noised and reference maps was utilized as the performance metric. As we report absolute E2A values in our study, we also report MAE for the E2A map. In all subsequent experiments, the reported metrics represent the mean values across all voxels within the left ventricular myocardium, excluding background and right ventricular regions. Throughout this study, HA and E2A, along with their associated error metrics, are presented in degrees. FA and its MAE are dimensionless quantities, while MD and its MAE are expressed in units of $10^{-3}\frac{mm^{2}}{s}$.

#### 2.4.1 Statistical analysis

Given the inability to ensure normal distributions among test subjects, all results were treated as non-parametric. The Wilcoxon signed-rank test was employed for statistical comparisons unless otherwise specified. A significance level of 0.05 was adopted for all statistical tests. When reporting intersubject variability, results are presented as median [interquartile range].

### 2.5 Comparison of deep learning-based de-noising models

#### 2.5.1 Model evaluation strategy

Both retrospective and prospective evaluations were conducted of our proposed models. In the retrospective analysis, our WGUFx5 model was compared against two baselines: the classical least-squares approach (LLS) and the U-Net model from Ferreira et al. [28], using aggregate image-level metrics. Through an ablation study, the impact of each proposed enhancement was systematically evaluated: tensor-to-tensor training (T2T), channel normalization (CN), augmented T2T, UFormer backbone architecture, GAN training objective (WGUF), and ensemble learning.

To gain a more spatially detailed understanding of the performance differences between our proposed WGUFx5 model and the U-Net baseline [28], we also conducted a granular, patch-level error comparison, presented in Fig. 3. As shown in the figure caption, this analysis involves scatter plots where each point represents the average absolute error calculated over a 5 × 5 pixel patch for diffusion tensor metrics (MD, FA, HA, E2A). The error for the proposed model (y-axis) is plotted against the error for the baseline model (x-axis). This approach allows for visualization of local performance differences, with points falling below the identity line indicating superior performance of the proposed model within that specific patch. This granular analysis complements the overall image-level metrics by revealing finer spatial patterns in model errors.

#### 2.5.2 Prospective validation

The generalizability of our approach was validated through additional testing on prospective data that was acquired from 5 patients. For each patient, five equidistant slices from the base to the apex of the LV were collected, yielding 25 total prospective slices. This dataset was used to evaluate the performance of the three primary models (LLS, U-Net, and WGUFx5) against the metrics established in the retrospective study.

### 2.6 Clinical validation

#### 2.6.1 Preservation of known properties

To validate our models’ physiological accuracy, two established cDTI properties were examined: the transmural variation of helix angle from endocardium to epicardium [3], [36], [37], [38], [39], [40], and the distribution of fractional anisotropy across the myocardial wall [41]. For HA analysis, normalized transmural profiles were calculated from the LV center to each epicardial border pixel. For FA analysis, the myocardial wall was divided into three equal-width zones (endocardium, mesocardium, and epicardium), and the distribution of FA values across these regions was compared.

#### 2.6.2 Performance in the presence of pathology

To compare the performance of the models both in the presence and absence of cardiac pathology, the test set was divided into healthy volunteers and clinical patients. Error metrics (MAE and MAAE) were compared between these groups, and the statistical significance of performance differences was analyzed.

#### 2.6.3 Myocardial infarction characterization

To evaluate whether the proposed model preserves clinically relevant diagnostic features, we analyzed acute myocardial infarction (MI) cases from our test cohort that had corresponding late-gadolinium enhancement (LGE) images. Previous studies have established that infarcted regions, identifiable by increased gadolinium concentration in LGE images, typically exhibit higher MD values compared to healthy (remote) regions [42].

For quantitative analysis, we

1. Used LGE images to identify infarcted and remote regions in the myocardium
2. Generated MD maps using LLS, U-Net, and WGUFx5 models across all breath-hold regimes (5BH, 3BH, 1BH)
3. Calculated mean MD values for both infarcted and remote regions
4. Computed the difference between infarcted and remote region MD values (ΔMD) for each model and compared it to the reference full-repetition acquisition

Statistical significance was assessed between infarcted and remote regions for each model. Additionally, we evaluated whether the ΔMD values from each model differed significantly from the reference acquisition’s ΔMD.

#### 2.6.4 Analysis of situs inversus totalis cases

To specifically test our model’s robustness to extreme anatomical variations, we evaluated its performance on patients with Situs Inversus Totalis (SIT), a rare condition where the heart and other organs are mirror-imaged compared to their normal positions. This mirroring extends to the cardiac microstructure, resulting in a partially reversed helical pattern of cardiomyocytes in the left ventricle [8]. Notably, no SIT cases were included in either the training or validation datasets, making this a true test of out-of-distribution generalization.

Given that SIT primarily affects the orientation of cardiac fibers, we focused our analysis on Helix Angle maps, where the impact of the condition is most pronounced. We generated HA maps using multiple approaches: LLS with full repetitions (10BH), and LLS, U-Net, and our proposed method with reduced repetitions. The maps were evaluated for anatomical plausibility and preservation of the characteristic reversed helical pattern, with quantitative error analysis performed between methods.

## 3 Results

### 3.1 Comparison of deep-learning-based de-noising models

#### 3.1.1 Retrospective study

**Fig. 2:**
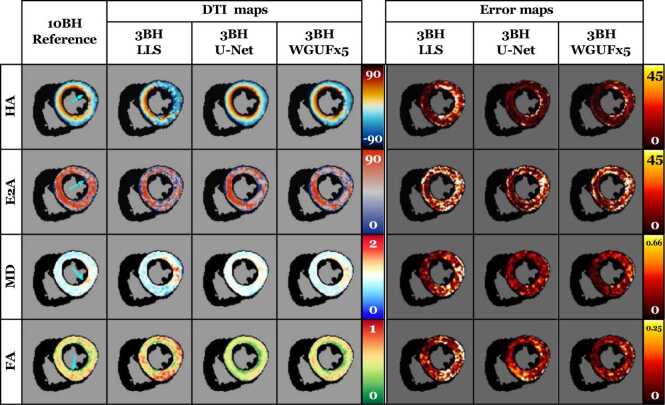
cDTI maps for an example from the test dataset. The tensors were computed using LLS, the U-Net model, and WGUFx5 (ours). The reference maps generated from all available data are compared to maps computed from the 3BH datasets. Additional qualitative results for 5BH and 1BH can be found in Appendix A. The absolute error maps are also displayed. Units: HA and E2A degrees, MD $10^{-3}\frac{mm^{2}}{s}$. *cDTI* cardiac diffusion tensor imaging, *LLS* linearized least squares, *MD* mean diffusivity, *HA* Helix Angle, *BH* breath holds, *WGUF* Wasserstein GAN UFormer, *E2A* sheetlet angle

Table 2 shows the performance comparison across all evaluated models. Our proposed WGUFx5 model, which combines all enhancements with a UFormer de-noising architecture, achieved significant noise reduction compared to both the LLS approach and the U-Net model (UN). The improvements were particularly pronounced in the 3BH dataset, where WGUFx5 reduced HA errors to 11 ± 6^∘^ compared to 15 ± 7^∘^ for LLS and 13 ± 5^∘^ for U-Net while maintaining comparable performance in E2A measurements (21 ± 8^∘^ vs 25 ± 7^∘^ for LLS). Specifically, our WGUFx5 model achieved over 20% reduction in cDTI error metrics compared to the U-Net baseline across the clinically relevant 3BH dataset, with improvements ranging from 10.5% (E2A, 21.2 vs 23.7) to 22.2% (FA, 0.07 vs 0.09), as detailed in Table 2. The visual comparison between the three primary models can also be seen in Fig. 2.

**Table 2:** cDTI maps errors for all the studied models. The table is split into three sections, one for each dataset. The best and second-best values for each metric and dataset are shown in bold and underlined, respectively. The symbol “” denotes an ablation study. The proposed model has lower de-noising errors than both LLS and Ferreira et al.’s [28] U-Net model by a significant margin for all metrics and datasets. The results with a non-significant difference with the LLS result are highlighted in red. Units: HA and E2A degrees, MD $10^{-3}\frac{mm^{2}}{s}$, FA dimensionless^a^.

a Data are reported as median [interquartile range].

In the ablation analysis, channel normalization demonstrated meaningful improvements over the standard U-Net model, particularly with higher-repetition datasets, reducing FA error in 5BH from 0.072 ± 0.03 to 0.070 ± 0.03. The subsequent addition of tensor-to-tensor training produced mixed results: while it significantly improved HA measurements in 3BH from 12 ± 4^∘^ to 11 ± 5^∘^, it led to increased MD errors (0.11 ± 0.05 × 10^−3^*mm*^2^*s*^−1^ vs 0.12 ± 0.06 × 10^−3^*mm*^2^*s*^−1^). The integration of data augmentation proved crucial, with UN + CN + aug-T2T achieving some of the strongest results among intermediate models, as evidenced by the reduction in E2A error from 24 ± 8^∘^ to 21 ± 8^∘^ in 3BH, outperforming both its predecessors and several more complex architectures.

**Fig. 3:**
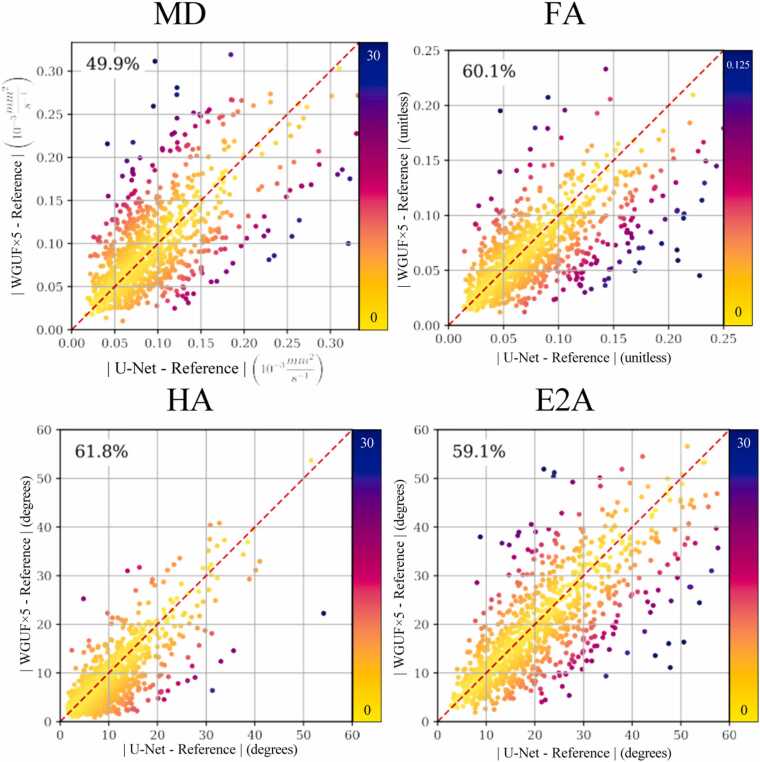
Patch-level error comparison between the proposed WGUFx5 model (y-axis) and the U-Net model [28] (x-axis) for MD, FA, HA, and E2A metrics for the 3BH dataset. Each point represents the average absolute error over a 5 × 5 patch relative to the reference. Points below the identity line (y=x, dashed red) indicate superior performance by the proposed model for that patch. Percentages indicate the fraction of patches where the proposed model’s error was lower than the baseline. Point color relates to the distance from the identity line. Units: HA and E2A degrees, MD $10^{-3}\frac{mm^{2}}{s}$. *WGUF* Wasserstein GAN UFormer, *MD* mean diffusivity, *FA* fractional anisotropy, *HA* Helix Angle, *E2A* sheetlet angle

The granular comparison via patch-level errors for the 3BH dataset (Fig. 3) provides further insight into the relative performance of the WGUFx5 and U-Net models. For the FA, HA, and E2A metrics, the proposed WGUFx5 model exhibited lower absolute error compared to the U-Net baseline in a majority of the 5 × 5 patches analyzed: 60.1% for FA (608/1012), 61.8% for HA (625/1012), and 59.1% for E2A (598/1012). In the case of the MD metric, the performance was closely matched, with the WGUFx5 model showing lower error in 49.9% of the patches (496/1012), indicating the U-Net baseline performed slightly better more often on a patch-by-patch basis for this specific metric within the 3BH dataset. The scatter plots visually confirm these percentages, with a higher density of points below the identity line observed for FA, HA, and E2A compared to MD, particularly in the regions representing lower absolute errors for both models.

#### 3.1.2 Prospective study

The prospective evaluation results, presented in Table 3, demonstrate that WGUFx5 maintains superior performance compared to both LLS and U-Net across all metrics. For the 5BH scenario, WGUFx5 achieved significantly lower errors in both helix angle (8 ± 1^∘^ vs 11 ± 4^∘^ for U-Net) and E2A (14 ± 3^∘^ vs 19 ± 5^∘^ for U-Net), while also showing consistently tighter distributions with lower interquartile ranges. This performance advantage was maintained even with reduced acquisitions. In the 3BH case, WGUFx5 showed improvements across all maps, with particularly notable reductions in HA (11 ± 3^∘^ vs 13 ± 5^∘^ for U-Net) and E2A (19 ± 5^∘^ vs 23 ± 6^∘^ for U-Net), again with smaller interquartile ranges indicating more consistent performance. This robust improvement in both accuracy and consistency in a prospective acquisition suggests strong generalization capabilities of our proposed model.

**Table 3:** cDTI maps errors for the main considered models for the prospective dataset. The table is split into three sections, one for each dataset (1BH, 3BH, 5BH). In bold best results for each metric and dataset. The results with a non-significant difference with the LLS result are highlighted in red. Units: HA and E2A degrees, MD $10^{-3}\frac{mm^{2}}{s}$, FA dimensionless^a^.

a Data are reported as median [interquartile range].

### 3.2 Clinical validation

#### 3.2.1 Preservation of known properties

**Fig. 4:**
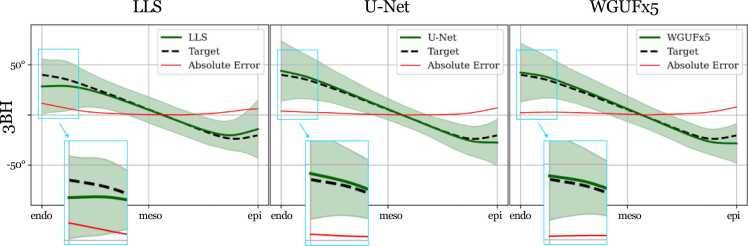
Profiles of intersubject median HA intramural lines from endocardium to epicardium for the 3BH dataset. The line profiles are calculated by ignoring the LV blood pool and moving from the center of the LV cavity to each epicardial border pixel. Each line profile is normalized in length before calculating the median line profile for each subject. The shaded area in the plot represents the standard deviation of all subjects’ mean line profiles in the test dataset, whereas the central line represents the intersubject mean. Absolute errors between the model and the target are also reported. *HA* Helix Angle, LV left ventricle, *LLS* linearized least squares, *WGUF* Wasserstein GAN UFormer, *E2A* sheetlet angle

The transmural HA profiles (Fig. 4) reveal distinct patterns across models. Both U-Net and WGUFx5 broadly maintain the expected transmural variation, with errors mainly concentrated at the epicardial region, where they underestimate HA values. LLS shows larger deviations, overestimating epicardial HA while underestimating endocardial values. The zoomed-in regions highlight that WGUFx5 adheres more closely to the target curve in the endocardial region compared to both U-Net and LLS.

**Fig. 5:**
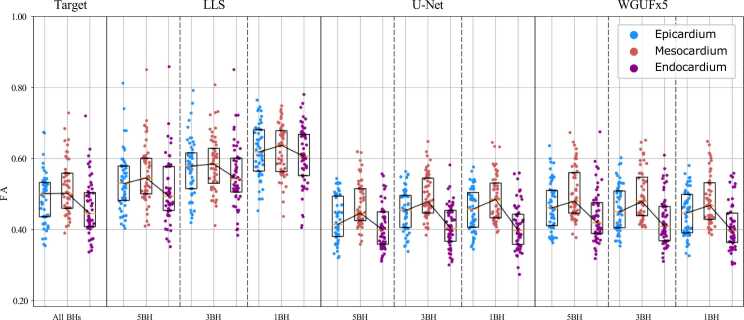
FA values (as percentages) for the endocardium, mesocardium, and epicardium. The three transmural zones were created by dividing the myocardial thickness into three equal-width zones around the entire myocardial ring. Each dot indicates the median FA from the test dataset for one individual in the corresponding transmural area. The median (yellow line) and interquartile range are also displayed for each column (black rectangles). A line connects the median values of the three zones. *FA* fractional anisotropy,*LLS* linearized least squares, *WGUF* Wasserstein GAN UFormer

FA distributions (Fig. 5) demonstrate the expected higher mesocardial values across models, though our ground-truth data show nearly identical median values for epicardium and mesocardium with higher variability in the mesocardium. LLS results show increasing FA values as breath-holds decrease, reaching values above 0.6 in the 1BH dataset. Both deep learning models adequately maintain the expected tissue property and show greater stability across different breath-hold regimes.

#### 3.2.2 Performance in the presence of pathology

All models showed consistently higher errors when processing clinical cases compared to healthy volunteers, with these differences being statistically significant across all metrics and breath-hold regimes, as shown in Table 4. In the 5BH dataset, WGUFx5′s HA error increased from 6 ± 4^∘^ in healthy cases to 8 ± 3^∘^ in clinical cases, while MD error increased from 7 ± 3 × 10^−3^*mm*^2^*s*^−1^ to 9 ± 5 × 10^−3^*mm*^2^*s*^−1^.

**Table 4:** cDTI maps errors (MAE and MAAE) for the main considered deep-learning models compared to the LLS model. The errors are grouped into errors on healthy volunteers and errors on clinical scans. Each value is colored red if the difference between healthy and patient subjects (same model, same metric, same dataset) is not significant. Units: HA and E2A degrees, MD $10^{-3}\frac{mm^{2}}{s}$, FA dimensionless^a^.

Healthy				Patient			
		LLS	WGUFx5			LLS	WGUFx5
HA	5BH	7.2 [5.1]	6.3 [3.9]	HA	5BH	10.2 [3.9]	8.5 [3.4]
	3BH	12.4 [7.4]	9.3 [4.8]		3BH	15.8 [6.8]	11.2 [5.0]
	1BH	15.6 [7.0]	10.5 [4.5]		1BH	21.6 [7.9]	12.3 [4.3]
E2A	5BH	13.3 [6.0]	11.8 [4.9]	E2A	5BH	19.1 [5.7]	17.0 [4.8]
	3BH	19.7 [9.1]	15.7 [7.5]		3BH	26.5 [4.7]	22.4 [6.0]
	1BH	26.2 [9.2]	17.7 [9.1]		1BH	32.5 [5.0]	23.9 [6.2]
MD	5BH	0.07 [0.04]	0.07 [0.03]	MD	5BH	0.10 [0.04]	0.09 [0.05]
	3BH	0.11 [0.05]	0.08 [0.05]		3BH	0.15 [0.04]	0.12 [0.05]
	1BH	0.17 [0.09]	0.09 [0.08]		1BH	0.25 [0.07]	0.13 [0.07]
FA	5BH	0.05 [0.03]	0.05 [0.02]	FA	5BH	0.08 [0.03]	0.06 [0.03]
	3BH	0.10 [0.06]	0.06 [0.03]		3BH	0.12 [0.04]	0.08 [0.03]
	1BH	0.13 [0.08]	0.07 [0.04]		1BH	0.18 [0.06]	0.09 [0.04]

*cDTI* cardiac diffusion tensor imaging, *MAE* mean absolute error, *MAAE* mean absolute angle error, *LLS* linearized least squares, *HA* Helix Angle, *MD* mean diffusivity, *FA* fractional anisotropy,*WGUF* Wasserstein GAN UFormer

a Data are reported as median [interquartile range].

This performance gap widened substantially as the number of breath-holds decreased. In the 1BH dataset, HA errors for WGUFx5 rose from 11 ± 5^∘^ to 12 ± 4^∘^ between healthy and clinical cases, while for LLS, the difference was more pronounced (16 ± 7^∘^ to 22 ± 8^∘^). Similar patterns were observed across all metrics, with E2A showing particularly large differences in the 1BH scenario (26 ± 9^∘^ vs 33 ± 5^∘^ for LLS). Despite these increases, WGUFx5 maintained consistently lower errors than LLS across both populations and all breath-hold regimes, demonstrating better resilience to pathological variations.

#### 3.2.3 Myocardial infarction characterization

The reference acquisitions showed mean MD values of 1.25 ± 0.20 × 10^−3^*mm*^2^*s*^−1^ for infarcted regions and 1.05 ± 0.19 × 10^−3^*mm*^2^*s*^−1^ for remote regions, yielding a reference ΔMD of 0.22 ± 0.13 × 10^−3^*mm*^2^*s*^−1^. For all models and breath-hold regimes, two key observations emerged as follows: 1) the difference between infarcted and remote region MD values remained statistically significant, and 2) none of the models’ ΔMD values differed significantly from the reference ΔMD.

In the 5BH regime, LLS achieved a ΔMD of 0.20 ± 0.20 × 10^−3^*mm*^2^*s*^−1^, followed closely by U-Net (0.19 ± 0.13 × 10^−3^*mm*^2^*s*^−1^) and WGUFx5 (0.18 ± 0.17 × 10^−3^*mm*^2^*s*^−1^). For 3BH reconstructions, U-Net showed slightly higher contrast (ΔMD = 0.24 ± 0.10 × 10^−3^*mm*^2^*s*^−1^), while WGUFx5 maintained more stable performance (0.17 ± 0.13 × 10^−3^*mm*^2^*s*^−1^). The 1BH regime saw slightly reduced but still significant contrast across all methods (LLS: 0.19 ± 0.21 × 10^−3^*mm*^2^*s*^−1^, U-Net: 0.15 ± 0.15 × 10^−3^*mm*^2^*s*^−1^, WGUFx5: 0.16 ± 0.14 × 10^−3^*mm*^2^*s*^−1^).

**Fig. 6:**
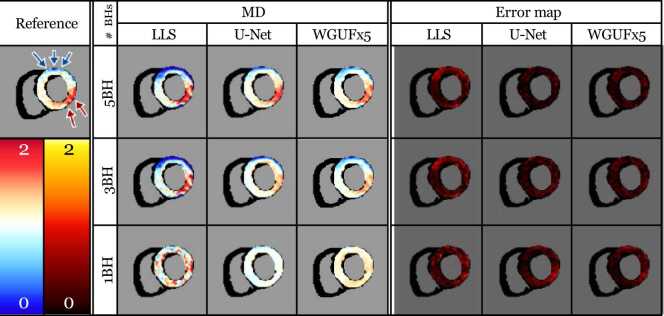
An example of MD maps and error maps for a clinical patient. The red arrows indicate the infarcted region, while the blue arrows indicate the remote region, as determined by the presence of gadolinium in a late-gadolinium enhancement scan. For this comparison, only the LLS, U-Net, and WGUFx5 models are used. Units: MD $10^{-3}\frac{mm^{2}}{s}$. *MD* mean diffusivity, *LLS* linearized least squares, *WGUF* Wasserstein GAN UFormer

The MD maps (Fig. 6) provide visual confirmation of these quantitative findings. The reference image shows clear differentiation between infarcted (red arrow) and remote (blue arrow) regions. This contrast remains well-preserved in 5BH reconstructions across all models, with minimal error in the corresponding error maps. The 3BH reconstructions maintain this distinction, though with slightly increased error, particularly in the deep learning approaches. At 1BH, while overall structural features are preserved, the contrast between infarcted and remote regions becomes less pronounced, especially in the U-Net and WGUFx5 reconstructions, as evidenced by increased values in the error maps.

#### 3.2.4 Analysis of situs inversus totalis cases

**Fig. 7:**
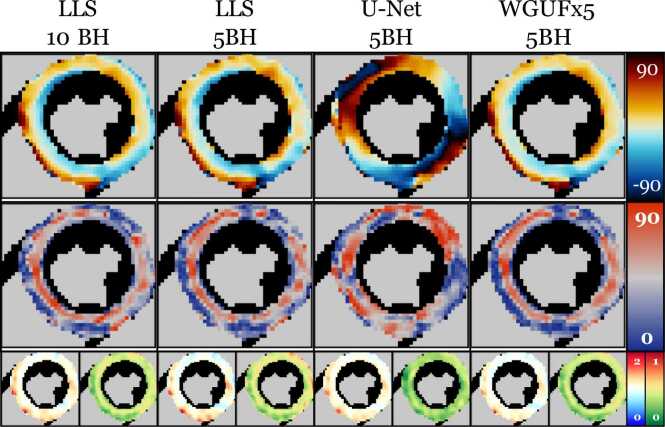
cDTI maps from a SIT patient using different methods. From left: LLS 10BH, LLS 5BH, U-Net 5BH, and proposed method 5BH. The last three columns represent reduced-repetition (5BH) reconstructions. While U-Net is mostly unable to recover the correct micro-structure, LLS and WGUFx5 produce plausible results, with WGUFx5 showing the best visual fidelity. Units: HA and E2A degrees, MD $10^{-3}\frac{mm^{2}}{s}$. *cDTI* cardiac diffusion tensor imaging, *SIT* Situs Inversus Totalis, *LLS* linearized least squares, *HA* Helix Angle, *WGUF* Wasserstein GAN UFormer

The analysis of SIT cases revealed significant differences in model performance. The reference 10BH LLS reconstruction (Fig. 7) clearly shows the characteristic reversed helical pattern of SIT, with positive angles (red) in regions typically showing negative angles in normal hearts, and vice versa.

When reducing the number of breath-holds to 5, the LLS method showed degraded results with poor anatomical detail, though the general reversed pattern remained visible. The U-Net approach completely failed due to domain shift, generating anatomically implausible fiber patterns with no resemblance to the unique SIT structure. In contrast, WGUFx5 successfully preserved the reversed helical pattern even with reduced data (5BH), maintaining both structural integrity and distinctive SIT features. Table 5.

**Table 5:** Mean MD values for MI-affected patients’ infarcted (inf) and remote (rem) LV regions. The per-patient mean difference between average MD in the infarcted region and in the remote region reported as ΔMD. The difference in values between each “inf” and “rem” pairs is significant for all models and none of the ΔMD are significantly different compared to the reference acquisition’s ΔMD. Units: MD $10^{-3}\frac{mm^{2}}{s}$^a^.

		5BH	3BH	1BH
Reference	inf	1.25 [0.20]	1.25 [0.20]	1.25 [0.20]
	rem	1.05 [0.19]	1.05 [0.19]	1.05 [0.19]
	ΔMD	0.22 [0.13]	0.22 [0.13]	0.22 [0.24]
LLS	inf	1.22 [0.21]	1.24 [0.23]	1.28 [0.24]
	rem	1.07 [0.28]	1.01 [0.34]	1.11 [0.26]
	ΔMD	0.20 [0.20]	0.18 [0.16]	0.19 [0.21]
U-Net	inf	1.26 [0.18]	1.24 [0.23]	1.18 [0.17]
	rem	1.07 [0.21]	1.03 [0.23]	1.04 [0.11]
	ΔMD	0.19 [0.13]	0.24 [0.10]	0.15 [0.15]
WGUFx5	inf	1.23 [0.20]	1.18 [0.18]	1.19 [0.22]
	rem	1.06 [0.25]	0.97 [0.27]	1.07 [0.19]
	ΔMD	0.18 [0.17]	0.17 [0.13]	0.16 [0.14]

*MD* mean diffusivity*, MI* myocardial infarction*, LV* left ventricle*, LLS* linearized least squares*, BH* breath holds, *WGUF* Wasserstein GAN UFormer

a Data are reported as median [interquartile range].

Quantitative analysis revealed striking differences in model performance (Table 6). The U-Net completely breaks down due to domain shift, with HA errors remaining consistently high across all breath-hold regimes (49 ± 7^∘^, 48 ± 8^∘^, and 46 ± 8^∘^ for 1BH, 3BH, and 5BH, respectively). In contrast, our proposed method achieved substantially lower errors (21 ± 12^∘^, 15 ± 10^∘^, and 9 ± 8^∘^), demonstrating robust generalization to this unseen anatomical variation. Notably, our method even outperformed the classical LLS approach (21 ± 13^∘^, 16 ± 12^∘^, and 12 ± 9^∘^), indicating a minimal bias towards normal cardiac anatomy despite being trained exclusively on non-SIT cases.

**Table 6:** cDTI maps errors for the main considered models for the SIT dataset. The table is split into three sections, one for each dataset (1BH, 3BH, 5BH). Units: HA and E2A degrees, MD $10^{-3}\frac{mm^{2}}{s}$, FA dimensionless^a^.

	Model	HA	E2A	MD	FA
1BH	WGUFx5 + CN + aug-T2T	20.7 [11.6]	29.8 [9.1]	0.12 [0.09]	0.14 [0.06]
	U-Net	48.8 [6.5]	47.6 [6.6]	0.14 [0.10]	0.19 [0.05]
	Least squares (LLS)	20.6 [13.2]	30.9 [7.9]	0.18 [0.07]	0.15 [0.06]
3BH	WGUFx5 + CN + aug-T2T	15.3 [10.0]	26.1 [10.0]	0.09 [0.06]	0.11 [0.05]
	U-Net	48.4 [7.8]	45.8 [4.5]	0.09 [0.05]	0.17 [0.05]
	Least squares (LLS)	16.5 [12.3]	25.8 [9.1]	0.11 [0.11]	0.11 [0.08]
5BH	WGUFx5 + CN + aug-T2T	9.3 [8.0]	17.7 [8.5]	0.08 [0.06]	0.07 [0.03]
	U-Net	46.0 [8.5]	46.0 [8.5]	0.07 [0.05]	0.14 [0.06]
	Least squares (LLS)	11.6 [8.8]	18.1 [7.8]	0.07 [0.05]	0.08 [0.04]

*cDTI* cardiac diffusion tensor imaging, *SIT* situs inversus totalis, *HA* helix angle, *MD* mean diffusivity, *FA* fractional anisotropy, *CN* channel normalization, *LLS* late gadolinium enhancement,*WGUF* Wasserstein GAN UFormer

a Data are reported as median [interquartile range].

## 4 Discussion

### 4.1 Deep-learning-based de-noising

Our methodological modifications yielded significant improvements in tensor de-noising performance. The transition from image-to-tensor to tensor-to-tensor transformation revealed that decoupling the mathematical tensor computation from the de-noising process produces superior results. This finding suggests that the conventional unified approach may have imposed unnecessary constraints on the optimization process.

The impact of channel normalization on model performance highlights a critical aspect in tensor processing: the importance of appropriate feature scaling when handling distinct tensor components. The six unique elements of the diffusion tensor exhibit substantially different value distributions, making normalization particularly beneficial for model convergence.

The introduction of the adversarial component in the GAN-based architecture, combined with the UFormer backbone, provided consistent improvement across all evaluated metrics, leading to noticeably enhanced map quality (qualitative example in Fig. 2). This suggests that the discriminator network helps enforce tensor consistency beyond simple error minimization. The subsequent addition of model ensembling further enhanced the robustness of these improvements, particularly in challenging low-repetition scenarios.

The implementation of ensemble learning demonstrated benefits beyond conventional variance reduction. The enhanced stability across different acquisition scenarios suggests the existence of multiple viable solutions in the tensor representation space. Furthermore, the efficacy of bagging as a regularization mechanism indicates that the inherent complexity of diffusion tensor de-noising may be effectively addressed through ensemble methods.

The patch-level analysis (Fig. 3) complements the aggregate results (Table 2), confirming WGUFx5 outperformed the U-Net baseline in most local regions for FA, HA, and E2A in the 3BH dataset. This suggests our model’s enhancements effectively preserve local anisotropy and orientation. Notably, patch-level performance for MD was nearly equivalent (49.9% WGUFx5). This divergence might reflect MD’s strong baseline performance for this specific metric. Nonetheless, this granular view showing consistent local advantages for key fiber architecture metrics reinforces the overall benefit of the WGUFx5 approach.

Finally, while the original UFormer was designed for photographic images, its architecture proved particularly suitable for our cardiac diffusion imaging task due to its ability to handle complex, multi-channel tensor data and its effectiveness in addressing the specific types of noise artefacts commonly found in cDTI acquisitions.

### 4.2 Clinical validation

#### 4.2.1 Preservation of known properties

The transmural patterns observed in both HA and FA maps reveal important insights about the behavior and limitations of different de-noising approaches. Prior research has established that FA values should be higher in the mesocardium compared to the ventricular walls [41]. While all methods preserve the general expected biological patterns, their deviations from ground truth highlight distinct algorithmic biases that merit careful consideration.

The HA profiles in Fig. 4 demonstrate that deep learning approaches (U-Net and WGUFx5) tend toward systematic underestimation at the epicardium, suggesting these models may struggle with complex tissue interfaces where partial-volume effects, imperfect registration, or other boundary artefacts are prevalent. In contrast, LLS’s opposing pattern of epicardial overestimation and endocardial underestimation reveals the limitations of classical tensor fitting approaches.

The FA distribution patterns in Fig. 5 reveal complementary insights about model behavior across different myocardial zones. The heightened variability observed in mesocardial FA values, even in ground truth data, suggests this region may be inherently more heterogeneous in its microstructural organisation. The fact that WGUFx5 preserves this variability while maintaining closer alignment with reference values than LLS indicates it has achieved a better balance between de-noising and feature preservation. Notably, LLS shows an increase in FA values as breath-holds decrease, consistent with the expected behavior of noise contamination in diffusion tensor estimation [43]. The deep learning approaches appear more robust to this effect, maintaining more stable FA values across different breath-hold regimes.

According to prior research, FA should increase closer to the mesocardium and decrease closer to the ventricular walls [41]. Our analysis, in Fig. 5, shows that our ground-truth data has nearly identical median values for epicardium and mesocardium with higher variability in the mesocardium. Nonetheless, all LLS results for all datasets show the expected pattern. U-Net and WGUFx5 findings demonstrate the same trend in a more pronounced way; additionally, whereas LLS results give larger FA values overall than the ground truth, U-Net and WGUFx5 values are more in line with the expected reference values. It’s important to notice that an increase in FA with a reduced number of breath-holds is consistent with greater effects of noise [43].

These findings suggest that deep learning methods can effectively mitigate noise-induced biases in accelerated cDTI acquisitions, though careful consideration must be given to their tendency to underestimate angular measures at tissue boundaries. This characterization of model-specific behaviors provides important context for the clinical interpretation of cDTI maps derived using different approaches.

#### 4.2.2 Performance in the presence of pathology

cDTI analysis of pathological hearts presents distinct challenges compared to imaging healthy volunteers, as evidenced by the systematically higher error metrics in patient data across all models (Table 4). This performance gap widens notably as the number of breath-holds decreases.

The reduced performance of deep learning models in clinical cases can be attributed to several interconnected factors. The training data distribution contains relatively few examples of each specific pathology, potentially biasing the learned representations toward healthy cardiac microstructure. However, WGUFx5 shows notably better performance than LLS across all metrics in both populations, with the advantage being particularly pronounced in clinical cases.

The performance degradation with reduced breath-holds follows different patterns in the two populations. While both groups show increased errors with fewer breath-holds, the effect is more pronounced in clinical cases. This asymmetric impact likely reflects the compounded effects of physiological challenges (such as irregular breathing patterns and potential motion artefacts) with reduced data acquisition.

Notably, even LLS, which applies no anatomical priors, shows this difference in performance, suggesting that the challenges in clinical imaging are caused by fundamental data quality differences rather than model-specific limitations. However, the sustained superior performance of WGUFx5 across both populations and all breath-hold regimes indicates that learned priors can effectively mitigate these challenges while preserving clinically relevant features.

#### 4.2.3 Myocardial infarction characterization

The maintenance of statistically significant MD contrast between infarcted and remote regions, even with substantial acceleration, demonstrates the potential for accelerated cDTI in clinical settings. However, the progressive reduction in contrast magnitude with fewer breath-holds reveals important practical constraints. While diagnostic differentiation remains possible even at 1BH, the diminished effect size could impact clinical confidence, particularly for subtle tissue changes. The 3BH regime emerges as a promising compromise in this scenario, offering meaningful acquisition time reduction while maintaining robust tissue characterization.

#### 4.2.4 Analysis of situs inversus totalis cases

The dramatic failure of U-Net in SIT cases, despite its strong performance in normal anatomy, highlights a critical vulnerability in conventional deep-learning approaches to cardiac imaging. This collapse reveals how strongly these architectures can encode anatomical assumptions, potentially creating dangerous blind spots in clinical deployment. The contrasting success of WGUFx5, achieving lower errors than even classical LLS methods, demonstrates how architectural choices can promote robust generalization without requiring explicit training on anatomical variants.

While our method uses LLS as input, the improvement over LLS in SIT cases is particularly noteworthy. This enhanced performance demonstrates that WGUFx5 performs genuine signal enhancement rather than imposing learned anatomical priors. Despite being trained exclusively on non-SIT cases, the model successfully preserves and enhances the reversed helical pattern characteristic of SIT, suggesting it has learned generalizable features of cardiac microstructure rather than specific anatomical configurations.

These findings are relevant for rare conditions where gathering sufficient training data is impractical. The ability to reliably handle unseen anatomical variations without specific training examples represents a significant advance toward clinical viability, suggesting that focusing on architectural robustness may be more productive than attempting to capture every possible anatomical variant in training data.

## 5 Limitations

Several limitations of this study should be acknowledged. First, while our dataset encompasses a wide range of cardiac pathologies, some conditions (e.g., hDCM with 4 cases, Marfan’s syndrome with 7 cases) are under-represented, which could affect model performance for these specific pathologies. Second, the current implementation requires manual delineation of the left ventricular myocardium, introducing an operator-dependent step in the workflow.

Third, our tensor estimation relied on a Linear Least Squares (LLS) method. While this choice ensures consistency with the initial processing pipeline, current practice increasingly favors more robust techniques like Weighted Least Squares (WLS) for their superior handling of noise. Adopting a more advanced fitting method could potentially improve performance across all models, as the tensor estimation is a foundational component of the entire workflow.

Fourth, the comparison involves our ensemble model (WGUFx5, combining five networks) versus a single-network baseline (U-Net [28]). This structure could be perceived as giving an inherent advantage to our approach purely through ensemble effects like variance reduction. While acknowledging this potential contribution to WGUFx5′s performance, it is worth noting that qualitative observations revealed instances where the single U-Net model exhibited specific failure modes in challenging scenarios such as SIT patients. This observation suggests that the performance gains may stem not only from ensemble variance reduction but also from the architectural ability to handle these fundamentally challenging cases. Fifth, although we demonstrated the preservation of clinically relevant features such as infarct characterization, additional validation may be needed for other specific clinical applications not examined in this study.

Finally, the training data for this work consists exclusively of STEAM acquisitions for cDTI. To probe the model’s generalization capabilities, we conducted a preliminary evaluation on a clinical spin-echo (SE) dataset, which was entirely unseen by the model during training. The results on this out-of-distribution data highlight a significant generalization challenge. As detailed in supplementary Table A1, the quantitative performance of our WGUFx5 model on the SE data was not only substantially lower than on the native STEAM data, but it also often underperformed the conventional LLS baseline. This outcome is consistent with the model having no prior exposure to SE acquisitions. Importantly, the model processed the data without catastrophic failure, establishing a crucial performance baseline and suggesting the architectural framework is fundamentally sound. Future work will focus on acquiring a dedicated SE dataset; by augmenting our current training data with these new acquisitions and retraining the model, we aim to incrementally close this performance gap, thereby enhancing the model’s generalization ability and broadening its clinical utility.

## 6 Conclusion

cDTI has the potential to contribute substantially to clinical CMR by uniquely characterizing the microstructural organisation of the myocardium. However, its long acquisition time limits its viability for clinical use. In this paper, we propose addressing the issue by lowering the number of cDTI repetitions, which can lower overall acquisition time but also decrease SNR. We also proposed numerous enhancements to existing deep learning models that, when applied simultaneously, resulted in increased SNR, enabling robust cDTI acquisition with 3–5 breath-holds, a significant advancement toward improved clinical efficiency and feasibility. While our method shows promise across different acquisition strategies, the most robust and clinically viable results were achieved with 3–5 breath-hold protocols, where the method consistently maintains diagnostic accuracy while providing meaningful scan time reduction. Our method’s ability to maintain accuracy even in challenging anatomical variants like Situs Inversus Totalis, where previous approaches completely fail, demonstrates that this is not merely an incremental improvement but a significant advance in robust clinical applicability. Finally, we thoroughly validated our technique by demonstrating that its de-noised output retains critical tissue features that have been associated with clinical utility. Future work will focus on utilizing these methods prospectively on large-scale cohorts in both volunteer and patient studies for clinical translation.

## Code availability statement

The code is available upon request.

## Declaration of generative AI in scientific writing

During the preparation of this work, the authors used Claude 3.5 Sonnet to proofread and make minor presentation adjustments (e.g., rephrasing individual sentences) after the draft was finalized. After using this tool/service, the author(s) reviewed and edited the content as needed and take(s) full responsibility for the content of the publication.

## Author contributions

Michael Tänzer Writing – review & editing, Writing – original draft, Visualization, Software, Methodology, Investigation, Formal analysis, Data curation, Conceptualization^a,^* (m.tanzer@imperial.ac.uk), Andrew D. Scott Writing – review & editing^b^, Zohya Khalique Data curation^b^, Maria Molto Data curation^b^, Ramyah Rajakulasingam Data curation^b^, Ranil De Silva Data curation^b^, Dudley J. Pennell Project administration, Funding acquisition^b^, Pedro F. Ferreira Writing – review & editing, Supervision, Methodology, Data curation, Conceptualization^a^, Guang Yang Writing – review & editing, Supervision, Methodology, Funding acquisition^a,^*^,1^, Daniel Rueckert Supervision, Funding acquisition^c,1^, Sonia Nielles-Vallespin Writing – review & editing, Supervision, Project administration, Methodology, Conceptualization.

## Declaration of competing interests

The authors declare that they have no known competing financial interests or personal relationships that could have appeared to influence the work reported in this paper.

## Figures and Tables

**Fig. A1:**
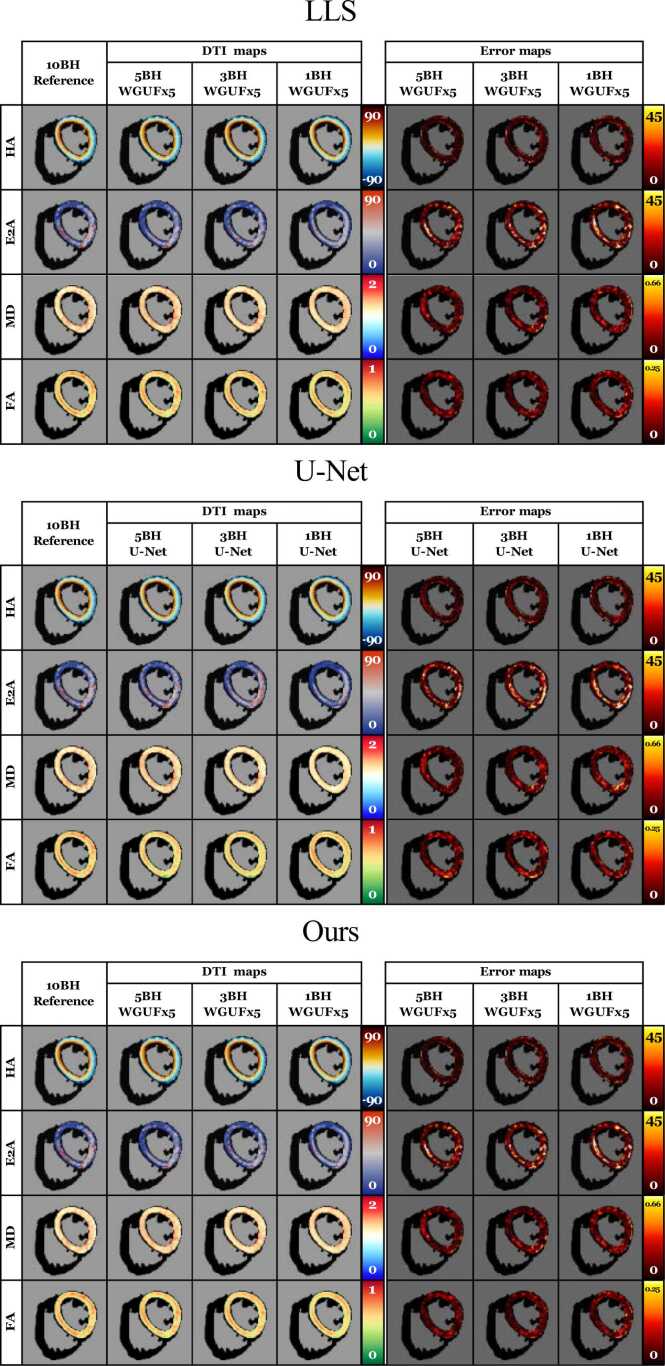
cDTI maps for the best example from the test dataset in terms of WGUFx5 test error. The tensors were computed using LLS, the U-Net model, and WGUFx5 (ours). The reference maps generated from all available data are compared to maps computed from the 5BH, 3BH, and 1BH datasets. The absolute error maps are also displayed. Units: HA and E2A degrees, MD $10^{-3}\frac{mm^{2}}{s}$.

**Fig. A2:**
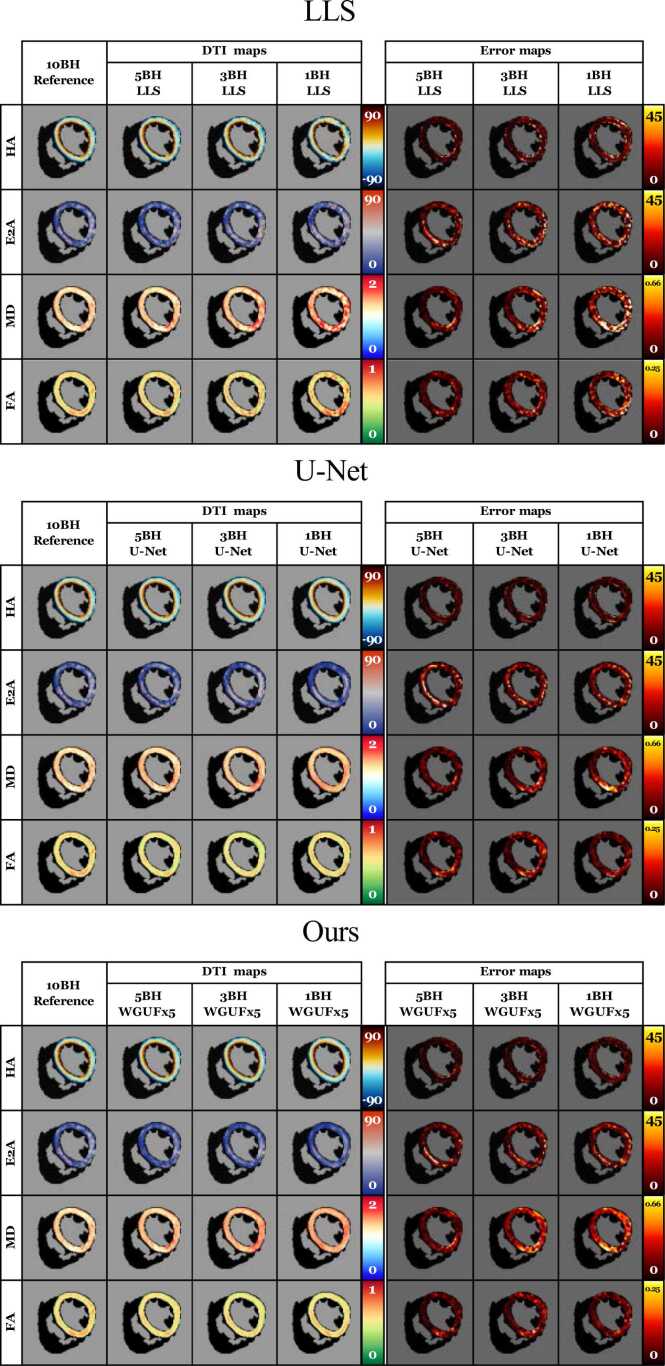
cDTI maps for the median example from the test dataset in terms of WGUFx5 test error. The tensors were computed using LLS, the U-Net model, and WGUFx5 (ours). The reference maps generated from all available data are compared to maps computed from the 5BH, 3BH, and 1BH datasets. The absolute error maps are also displayed. Units: HA and E2A degrees, MD $10^{-3}\frac{mm^{2}}{s}$.

**Fig. A3:**
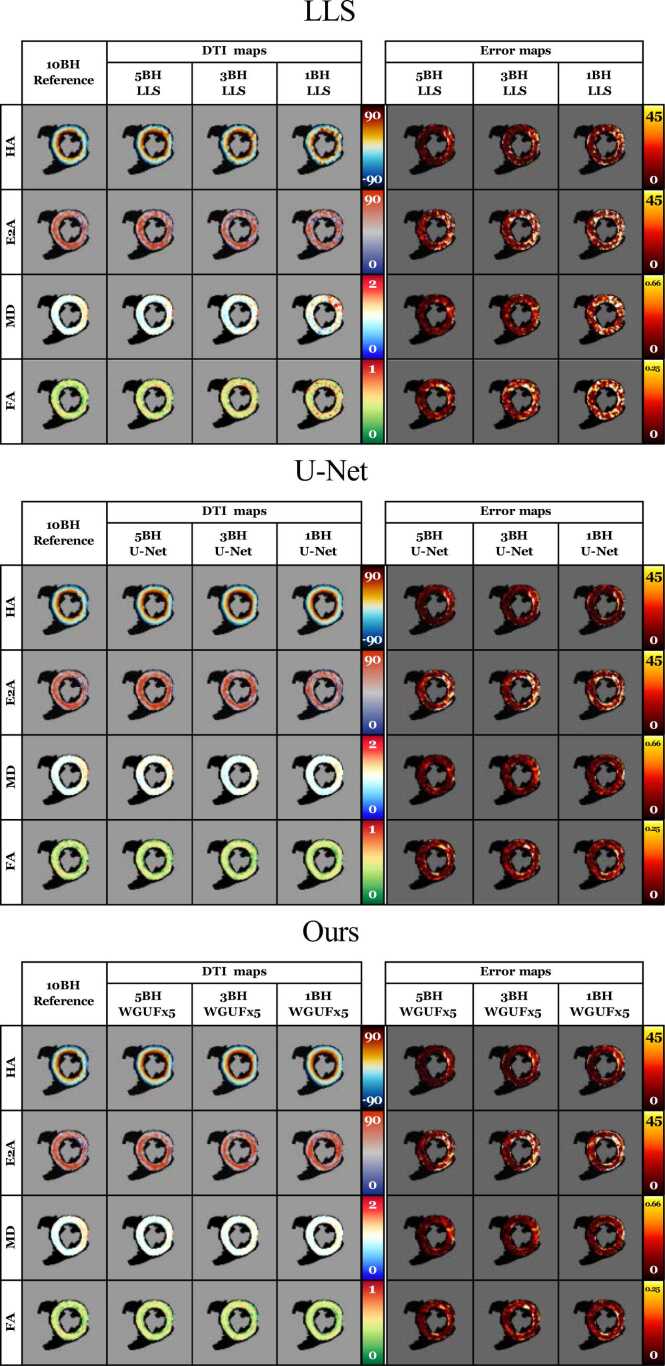
cDTI maps for the worst example from the test dataset in terms of WGUFx5 test error. The tensors were computed using LLS, the U-Net model, and WGUFx5 (ours). The reference maps generated from all available data are compared to maps computed from the 5BH, 3BH, and 1BH datasets. The absolute error maps are also displayed. Units: HA and E2A degrees, MD $10^{-3}\frac{mm^{2}}{s}$.

**Fig. A4:**
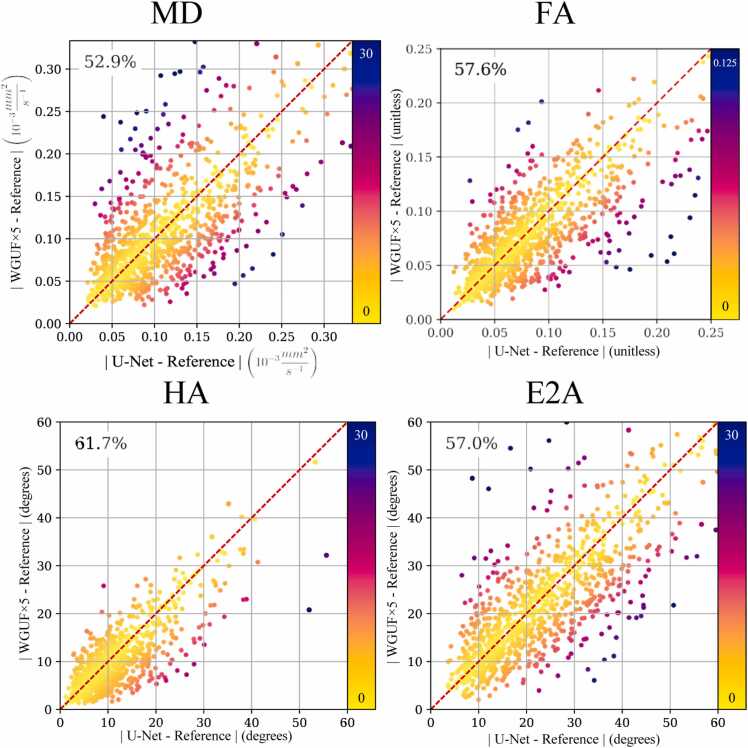
Patch-level error comparison between the proposed WGUFx5 model (y-axis) and the U-Net model [28] (x-axis) for MD, FA, HA, and E2A metrics for the 1BH dataset. Each point represents the average absolute error over a 5 × 5 patch relative to the reference. Points below the identity line (y=x, dashed red) indicate superior performance by the proposed model for that patch. Percentages indicate the fraction of patches where the proposed model’s error was lower than the baseline. Point color relates to the distance from the identity line. Units: HA and E2A degrees, MD $10^{-3}\frac{mm^{2}}{s}$.

**Fig. A5:**
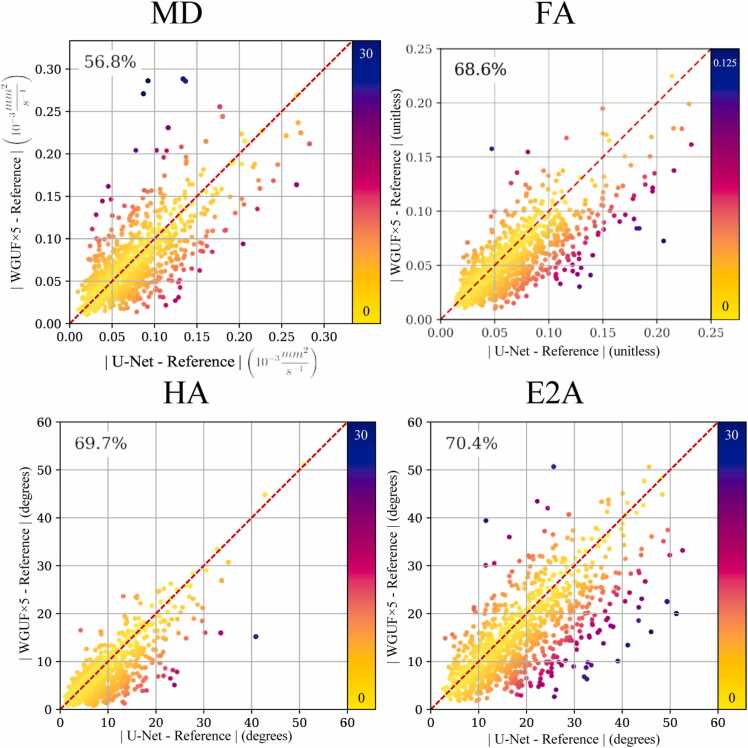
Patch-level error comparison between the proposed WGUFx5 model (y-axis) and the U-Net model [28] (x-axis) for MD, FA, HA, and E2A metrics for the 5BH dataset. Each point represents the average absolute error over a 5 × 5 patch relative to the reference. Points below the identity line (y=x, dashed red) indicate superior performance by the proposed model for that patch. Percentages indicate the fraction of patches where the proposed model’s error was lower than the baseline. Point color relates to the distance from the identity line. Units: HA and E2A degrees, MD $10^{-3}\frac{mm^{2}}{s}$.

**Fig. A6:**
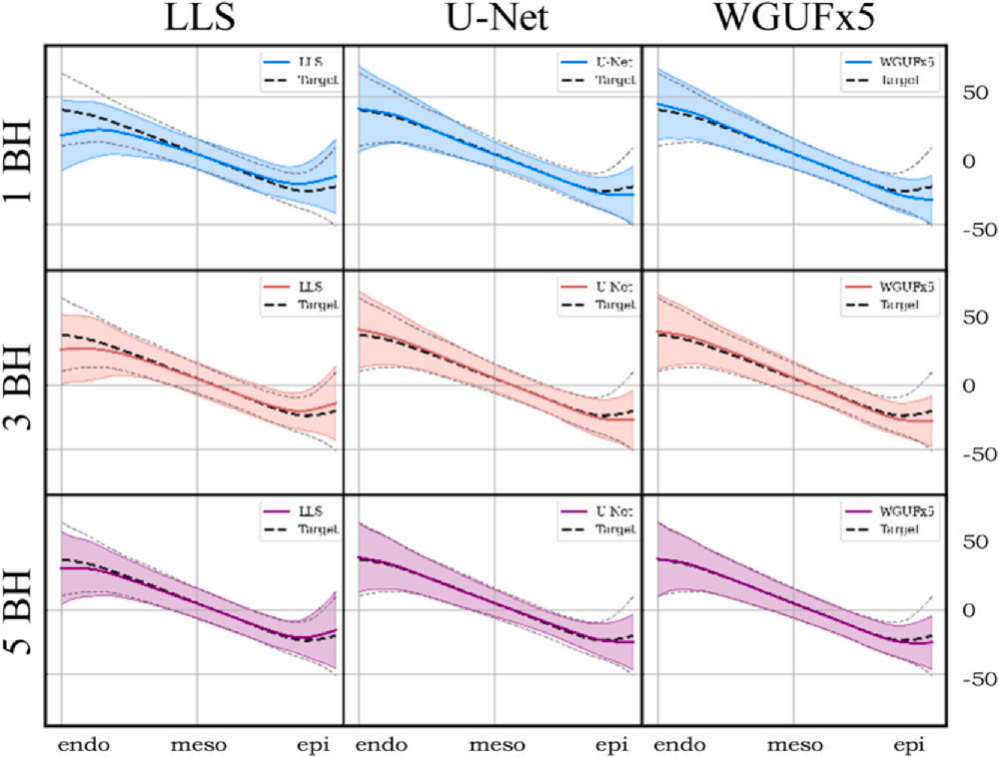
Profiles of intersubject median HA intramural lines from endocardium to epicardium. The line profiles are calculated by ignoring the LV blood pool and moving from the center of the LV cavity to each epicardial border pixel. Each line profile is normalized in length before calculating the median line profile for each subject. The shaded area in the plot represents the standard deviation of all subjects’ mean line profiles in the test dataset, whereas the central line represents the intersubject mean. *HA* Helix Angle, *LV* left ventricle

**Table A1:** Performance comparison between the WGUFx5 model and LLS on a spin-echo (SE) dataset comprised of 20 scans. This is designed to benchmark the model’s robustness on other acquisition sequences as WGUFx5 was not exposed to any SE data during its training phase. The table is split into three sections for 1, 3, and 5 breath-holds (BH). Units: HA and E2A degrees, MD $10^{-3}\frac{mm^{2}}{s}$, FA dimensionless.

	Model	HA	E2A	MD	FA
1BH	WGUFx5	13.51 [3.24]	32.92 [10.00]	0.17 [0.07]	0.11 [0.02]
	Least squares (LLS)	11.08 [3.59]	32.76 [5.76]	0.19 [0.06]	0.10 [0.03]

3BH	WGUFx5	10.76 [2.13]	29.08 [7.98]	0.17 [0.06]	0.12 [0.02]
	Least squares (LLS)	6.92 [1.98]	24.16 [5.46]	0.14 [0.04]	0.07 [0.02]

5BH	WGUFx5	8.21 [2.00]	27.6 [8.78]	0.15 [0.04]	0.12 [0.02]
	Least squares (LLS)	4.78 [1.82]	15.9 [4.66]	0.08 [0.07]	0.04 [0.01]

## Data Availability

The medical imaging datasets analyzed in this study cannot be made publicly available due to patient privacy restrictions and institutional ethics requirements that protect confidential patient information.

## Appendix A Additional qualitative results

Fig. A1.

Fig. A2.

Fig. A3.

Fig. A4.

Fig. A5.

## B HA transmural pattern between endocardium and epicardium

Fig. A6.

## C Model generalization to spin-echo data

Table A1.
